# Growth after pediatric and neonatal acute kidney injury: a meta-analysis

**DOI:** 10.1007/s00467-025-06801-6

**Published:** 2025-05-09

**Authors:** Michelle C. Starr, Mital Patel, Faizeen Zafar, Melissa S. Zhou, Russell Griffin, Annabel Biruete, Vedran Cockovski, Rasheed Gbadegesin, Dana Y. Fuhrman, Katja M. Gist, Cherry Mammen, Shina Menon, Catherine Morgan, Cara L. Slagle, Scott Sutherland, Michael Zappitelli, Danielle E. Soranno

**Affiliations:** 1 Division of Nephrology, Department of Pediatrics, Indiana University School of Medicine, 1044 West Walnut Street, Indianapolis, IN 46202 USA; 2 Division of Child Health Service Research, Department of Pediatrics, Indiana University School of Medicine, Indianapolis, IN USA; 3 Division of Pediatric Nephrology, Department of Pediatrics, Wake Forest University School of Medicine, Winston-Salem, NC USA; 4 Division of Nephrology, Department of Pediatrics, Duke University School of Medicine, Durham, NC USA; 5 Hospital for Sick Children, Toronto, ON Canada; 6 Department of Pediatrics, Cincinnati Children’s Hospital Medical Center, University of Cincinnati, Cincinnati, OH USA; 7 Division of Pediatric Nephrology, Department of Pediatrics, Stanford University, Palo Alto, CA USA; 8 Department of Epidemiology, University of Alabama at Birmingham, Birmingham, AL USA; 9 Department of Nutrition Science, Purdue University, West Lafayette, IN USA; 10 Division of Nephrology, Department of Medicine, Indiana University School of Medicine, Indianapolis, IN USA; 11 University of Pittsburgh Medical Center Children’s Hospital of Pittsburgh, Pittsburgh, PA USA; 12 Division of Critical Care, Children’s Hospital Colorado, Aurora, CO USA; 13 Division of Nephrology, Department of Pediatrics, University of British Columbia, Vancouver, Canada; 14 Department of Pediatrics, Division of Nephrology, University of Alberta, Edmonton, AB USA; 15 Division of Neonatology, Department of Pediatrics, Indiana University School of Medicine, Indianapolis, IN USA; 16 Weldon School of Bioengineering, Purdue University, West Lafayette, IN USA

**Keywords:** Acute kidney injury, Acute kidney failure, Growth, Neonates, Development

## Abstract

**Background:**

Acute kidney injury (AKI) occurs commonly in critically ill children. The impact of AKI on pediatric growth outcomes has been sparsely described.

**Objective:**

To compare growth in children with a history of AKI compared to those without AKI. We hypothesized that children with AKI would have worse growth compared to those without AKI.

**Data sources:**

A convenience sample of existing prospective and retrospective cohorts of children with AKI who had already collected or were able to collect data on growth parameters before and after an episode of AKI.

**Study eligibility criteria:**

There are < 5 studies in the published literature on growth in children with AKI. These investigators were contacted, and additional studies were added by contacting primary investigators of studies of childhood AKI in which data on growth parameters was able to be collected.

**Participants and interventions:**

Children from existing cohorts evaluating AKI (exposure) during childhood. Each included cohort had previously received local IRB approval per institutional guidelines. As our study was a meta-analysis and only used cohort-level data, no IRB approval was required for this report.

**Study appraisal and synthesis methods:**

Growth parameters (length and weight z-scores) before and after an episode of AKI were compared using a meta-means analysis. MOOSE guidelines were used. Data were pooled using a random-effects model. Hedges *g* was calculated, and Higgins *I*^2^ statistic was used to define variability due to between-cohort heterogeneity.

**Results:**

We included 3,586 children from 17 existing cohorts of AKI in various populations, including infants, children with cardiac disease, solid organ transplant and critically ill children without cardiac disease with follow-up from 12 months to 11 years after AKI. At most distant follow-up, those with AKI had lower length z-score than those without AKI (mean difference -0.37 [95%CI -0.52, -0.22, *p* < 0.001]) and lower weight z-score (mean difference of -0.29 [95%CI -0.43, -0.15, *p* < 0.001]). This difference was most striking in infants, as those with AKI had impaired growth (both length z-score and weight z-score) after AKI compared to those without AKI.

**Limitations:**

The analysis included only a convenience sample of observational cohorts of children, study selection could have been biased, and we did not evaluate the relationship between decreased kidney function (e.g., chronic kidney disease) after AKI in these cohorts and its relationship to poor growth.

**Conclusions and implications of key findings:**

This meta-analysis found that children with AKI have impaired growth after AKI. These findings were most striking in infants. We suggest focusing on growth outcomes in both clinical care and research investigating the impacts of AKI.

**Systematic review registration number:**

NA.

**Graphical abstract:**

**Figure:**
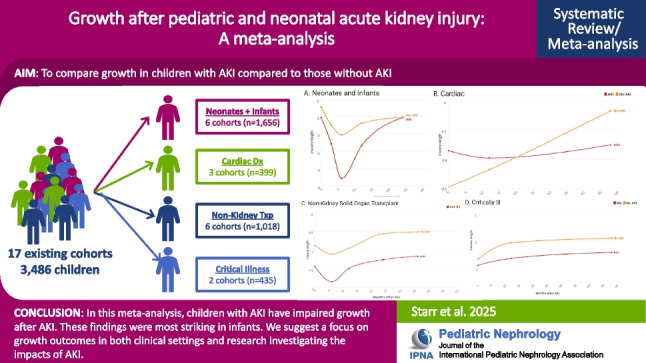
A higher resolution version of the Graphical abstract is available as Supplementary information

**Supplementary Information:**

The online version contains supplementary material available at 10.1007/s00467-025-06801-6.

## Introduction

Acute kidney injury (AKI) occurs commonly in critically ill patients, including infants and children [1, 2]. Children with AKI have increased length of stay, higher mortality, and, among those who survive to discharge, an increased risk of chronic kidney disease [3–6]. Preclinical models and emerging translational data suggest that AKI has far-reaching effects on many other major organ systems, including nutrition and growth [7, 8].

The association between AKI on long-term growth outcomes has been sparsely described in the pediatric literature [9, 10]. Macronutrient and micronutrient derangements, as well as alterations in growth factors, vitamins and minerals have been described in AKI [11]. AKI is a risk factor for negative protein and energy balance in critically ill children, which may be further worsened by losses in those receiving dialytic therapies [12]. Furthermore, in several animal studies from our group, we found slowed weight gain and differential length trajectories 1 year after AKI in young adult rodents [13, 14] and growth differences between AKI and sham pups 14 days after injury [15].

While growth is a paramount feature of early childhood health and development, the relationship between growth and AKI has been minimally investigated. Furthermore, our preclinical findings led us to question whether growth was impacted after episodes of pediatric AKI. To begin to fill this gap, we conducted a multicenter collaborative investigation, leveraging data from various institutions. We sought to compare growth in children with a history of AKI compared to those without AKI. We hypothesized that children with neonatal and childhood AKI would have worse growth (lower length-for-age and weight-for-age z-scores) compared to those without AKI.

## Methods

### Study selection and inclusion criteria

We included a convenience sample of existing prospective and retrospective cohorts of children with AKI who had already collected or were able to collect data on growth parameters before and after an episode of AKI (Table 1). Each cohort had been primarily designed to evaluate AKI and kidney outcomes but not growth. Each included cohort had previously received local IRB approval per institutional guidelines. As our study was a meta-analysis and only used cohort level data, no IRB approval was required for this report. IRB approval was obtained by each study (Table 1). We followed the Meta-analyses Of Observational Studies in Epidemiology (MOOSE) guidelines whenever possible (Supplemental Item 1) [16].

**Table 1:** Included cohorts and time of follow-up growth data

Author	Ref	N	Description	AKI Definition	Time of Follow-Up Growth Data							
					Baseline	Discharge	2–3 m	6 m	12 m	2 y	3 y	> 3 y
**Neonatal**												
Patel, et al	[17]	245	Premature neonates > 29 weeks	SCr, UOP	X		X	X	X			
Patel, et al	[17]	279	Neonates 29–36 weeks	SCr, UOP	X		X	X	X			
Starr, et al	[18, 19]	697	Extremely premature neonates from PENUT/REPaired Cohort [1, 20]	SCr		X				X		
Starr, et al	[20]	206	Neonates with BPD	SCr, UOP	X		X	X	X			
Starr, et al	[21]	189	Neonates with AKI or other neonatal kidney problem	SCr, UOP			X	X	X	X		
Slagle, et al	[22]	229	Neonates undergoing non-cardiac surgery	SCr, UOP	X		X	X	X			
**Cardiac**												
Morgan, et al	[23]	237	Infants undergoing Biventricular repair < 6 months	SCr	X					X		
Zappitelli, et al	-	51	Cardiac ICU age < 21 years	SCr	X							X
Gist, et al	[24, 25]	111	NEPHRON cohort at CCHMC, patient undergoing cardiac surgery as a neonate	SCr, UOP	X				X	X	X	
**Solid Organ Transplant**												
Menon, et al	[26]	67	Heart Transplant at age < 21 years	SCr	X			X	X	X	X	
Menon, et al	[26]	78	Liver Transplant at age < 21 years	SCr	X			X	X	X	X	
Fuhrman, et al	[27]	338	Solid organ non-kidney transplant at age < 19 years	SCr, UOP	X				X	X	X	
Morgan, et al	-	237	Heart Transplant at age < 21 years	SCr						X		
Zafar, et al	[28]	101	Heart Transplant at age < 21 years	SCr, UOP	X				X	X	X	
Zhou, et al	-	197	Heart Transplant at age < 21 years	SCr, UOP	X				X	X	X	
**ICU**												
Zappitelli, et al	[29, 30]	159	Cisplatin exposure at age < 21 years	SCr, UOP	X		X		X		X	
Zappitelli, et al	-	276	Pediatric ICU age < 21 years	SCr, UOP	X							X

*AKI,* acute kidney injury*;, BPD, *bronchopulmonary dysplasia*; ICU*, intensive care unit; *SCr*, serum creatinine; *UOP*, urine output

### Data extraction and definition of key variables

Each cohort defined AKI using serum creatinine Kidney Disease: Improving Global Outcomes (KDIGO) criteria [31]. In some of the cohorts, urine output criteria were also used (Table 1). The neonatal modification of the KDIGO criteria was used for cohorts of neonates and infants as their episode of AKI occurred during their index neonatal admission [31]. Height and weight were extracted from the electronic medical records at specified time points and transformed into z-scores to standardize and allow for group comparisons. Appropriate growth chart and representative z-scores based on the population, age, sex, and gestation of the child were used. For premature neonates < 37 weeks, the Fenton growth chart was used until 42 weeks postmenstrual age, followed by the World Health Organization (WHO) growth charts [32]. For infants born ≥ 38 weeks and children < 2 years, WHO growth charts were used. After 2 years of age, Centers for Disease Control and Prevention growth charts and z-scores were used [33].

### Statistical analysis

We first evaluated growth differences across all cohorts. However, as all subgroups were highly heterogeneous, we more closely evaluated growth differences within each subgroup (neonatal and infants, cardiac, non-kidney solid organ transplant and critically ill children) as the objective of our analysis was not to derive a universal estimate applicable to all patient subgroups but to identify and quantify an overarching signal of the association between AKI and growth across a range of pediatric populations. We chose these four subgroups as they are natural delineations in patient population and disease states as well as to decrease the heterogeneity of AKI exposure whenever possible. Despite this approach, we recognize that these populations remain heterogeneous. For each cohort, growth parameters were summarized with means and standard deviations for children with and without AKI, and data were compared using a meta-means analysis.

A random-effects model (Hartung-Knapp method) was used to calculate pooled estimates (Hedges *g*) and the 95% confidence interval (CI) to account for variations in the included cohorts. The random-effects model assumes that the true effect size may vary between studies and provides a weighted average that considers both within-study and between-study variance. Hedges *g* was calculated from each cohort’s means, SDs, and sample sizes. Cochran’s *Q* test was used to assess between-cohort heterogeneity. Higgins *I*^2^ statistic was used to define the percentage of variability due to between-cohort heterogeneity rather than sampling error. The *I*^2^ values of 25%, 50%, and 75% were taken to represent low, moderate, and high levels of heterogeneity, respectively.

Visual assessment of funnel plot symmetry and Egger’s and Begg’s tests were used to test for publication bias. Forest plots were generated to display standardized mean differences (SMDs) and their 95% CIs. We used Hedges and Olkin standard error for effect size. A two-sided *P*-value < 0.05 was considered statistically significant. All analyses and forest plots were generated using Stata/SE 18.0 (College Station, Texas). Summary figures were generated using Flourish (https://flourish.studio) and created in BioRender (https://BioRender.com) and incorporate standard data smoothing techniques for clarity.

## Results

### Overall cohort

We included a total of 3,586 children from 17 existing cohorts of AKI in various populations. These existing cohorts included neonates and infants (*N* = 6 cohorts [17–19, 21, 22]; 1,656 infants), children with cardiac disease (*N* = 3 cohorts [23, 34]; 399 children), solid organ transplantation (*N* = 6 cohorts [26–28]; 1,018 children), and critically ill children without cardiac disease (*N* = 2 cohorts [29, 30]; 325 children). Fourteen studies had baseline growth parameters at the time of or prior to an AKI episode. Timing of follow-up ranged from 12 months to 11 years after the index AKI event, depending on cohort and patient population (Table 1).

**Fig. 1:**
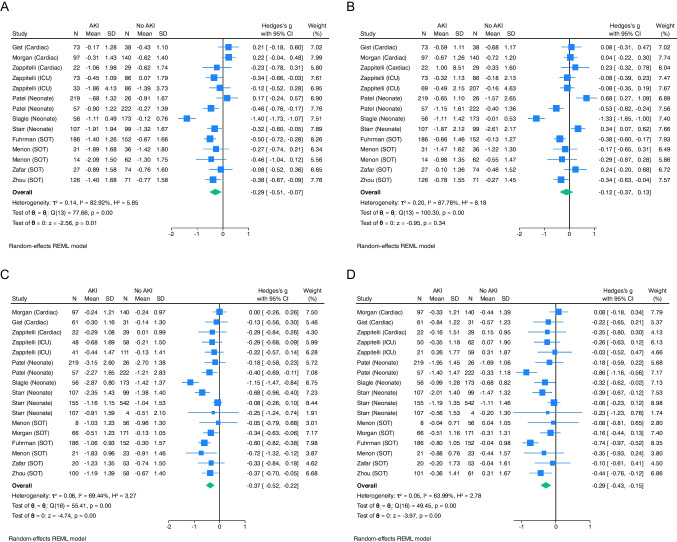
Forest plot of standardized mean differences and 95% CIs for growth z-scores for children and infants. Panels A and B show growth z-scores at the time of or prior to an AKI episode with length for age z-score (Panel **A**) and weight-for-age z-score (Panel **B**) in the 14 studies with baseline growth parameters. Panels C and D show growth z-scores at the time of most distant follow-up with length for age z-score (Panel **C**) and weight-for-age z-score (Panel **D**) for all 17 studies

We first compared growth parameters at baseline and at the most distant post-AKI follow-up across all cohorts. There was high heterogeneity (I^2^ = 83–88%) at baseline and moderate heterogeneity (I^2^ = 64–69) at the most distant follow-up. Of the 14 cohorts with baseline data available prior to or at the time of AKI episode, children and infants with an episode of AKI had lower length z-score than those without AKI (mean difference −0.29 [95% CI −0.51, −0.07, *p* = 0.01]). Children and infants with an episode of AKI did not have differences in weight z-score than those without AKI at baseline (mean difference in z-score of −0.12 [95% CI −0.37, 0.13, *p* = 0.34]) (Fig. 1A, B). Among all cohorts at the time of most distant follow-up, those with an episode of AKI had lower length z-score than those without AKI (mean length z-score difference −0.37 [95% CI −0.52, −0.22, *p* < 0.001]). Those with an episode of AKI also had lower weight z-score at the time of most distant follow-up (mean weight z-score difference of −0.29 [95% CI −0.43, −0.15, *p* < 0.001]) (Fig. 1C, D).

### Neonatal and infant cohorts

There were six cohorts included in the neonatal and infant meta-analysis, with 1,656 infants (*N* = 6, *n* = 1,656) (Table 1). These cohorts included infants admitted to the ICU (*N* = 3), those with bronchopulmonary dysplasia (*N* = 1), those with kidney insufficiency (e.g., AKI or delayed maturation of kidney function) (*N* = 1), and those undergoing non-cardiac surgery (*N* = 1) (Supplemental Item 2). Follow-up among the cohorts of neonates and infants ranged from 12 months of age (*N* = 4) to 24 months of age (*N* = 2). Among the four studies with baseline growth data prior to or at the time of AKI, there were no differences between groups regarding baseline length z-score with a mean difference of −0.51 (95% CI −1.15, 0.13, *p* = 0.12). There was no difference in baseline weight z-score between those with and without AKI with a mean difference of −0.21 (95% CI −1.09, 0.67, *p* = 0.64).

**Fig. 2:**
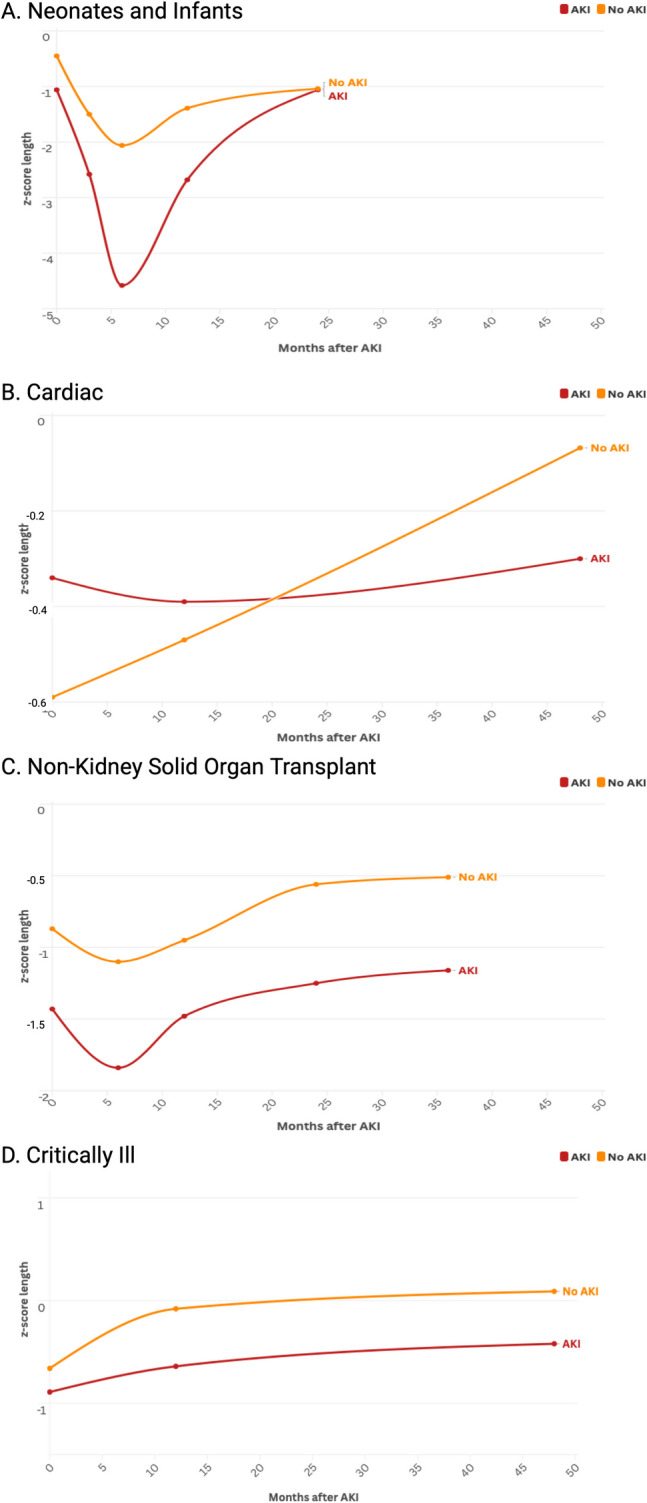
Length-for-age z-score following episode of AKI for neonates and infants (**A**), children with cardiac disease (**B**), children with non-kidney solid organ transplant (**C**), and critically ill non-cardiac disease (**D**) depicting meta-mean z-score in months following episode of AKI. In this figure, data points beyond 48 months are displayed at 48 months

**Fig. 3:**
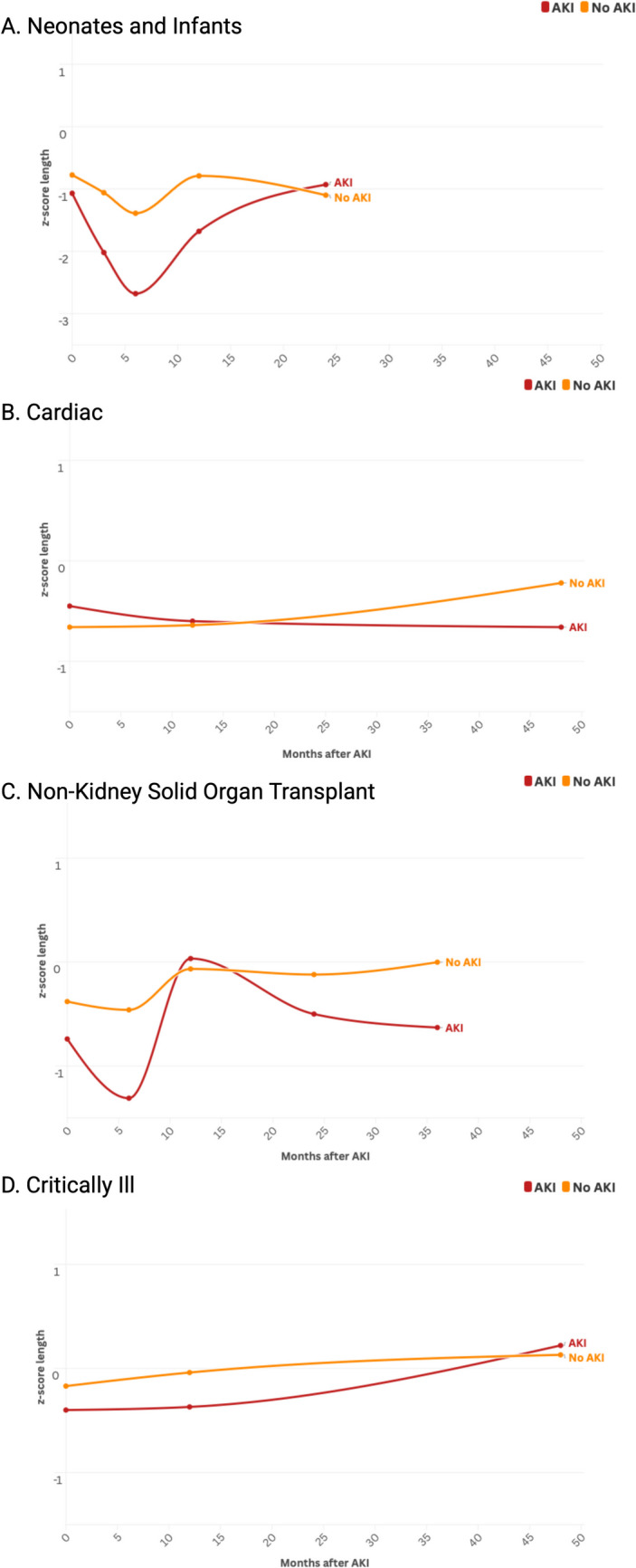
Weight-for-age z-score following episode of AKI for neonates and infants (**A**), children with cardiac disease (**B**), children with non-kidney solid organ transplant (**C**), and critically ill non-cardiac disease (**D**) depicting meta-mean z-score in months following episode of AKI. In this figure, data points beyond 48 months are displayed at 48 months

Infants with AKI had impaired growth (both length z-score and weight z-score) following their episode of AKI compared to those without AKI (Figs. 2 and 3A). Of the five studies with data available 2–3 months after the index AKI episode, infants with AKI had a lower length z-score than those without AKI (mean difference −0. [95%CI −0.63, −0.28, *p* < 0.001]). At 2–3 months of age, infants with an episode of AKI had a lower weight z-score than those without AKI (mean difference in z-score of −0.53 [95% CI −0.75, −0.32, *p* < 0.001]). Among the 5 studies with data available six months after AKI episode, infants with an episode of AKI had lower length z-score than those without AKI (mean length z-score difference −0.87 [95% CI −1.13, −0.60, *p* < 0.001]). Infants with an episode of AKI also had lower weight z-score at 6 months of age (mean weight z-score difference of −0.64 [95% CI −0.94, −0.33, *p* < 0.001]).

Growth differences persisted 12 months after the index AKI episode. Infants with an episode of AKI were shorter than those without AKI (mean length z-score difference −0.56 [95% CI −0.92, −0.19, *p* < 0.001]). Infants with an episode of AKI also weighed less 12 months after AKI (mean weight z-score difference of −0.43 [95% CI −0.69, −0.17, *p* < 0.001]). Only two studies had data available 24 months after the index AKI episode. There was no difference between infants with and without AKI with regards to height z-scores at 24 months (mean height z-score difference of −0.09 [95% CI −0.26, 0.09] *p* = 0.33), nor a difference in mean weight z-score at 24 months (mean weight z-score difference of −0.06 [95% CI −0.24, 0.11] *p* = 0.50). No sex differences were seen when results were analyzed unpooled regarding sex.

### Cardiac cohorts

Three cohorts were included in the cardiac subgroup (Table 1). These cohorts included a total of 399 children. We included cohorts with single ventricle physiology (*N* = 1), a single center cohort of children with neonatal cardiac surgery (*N* = 1), and a cohort of children admitted to the ICU with a primary cardiac diagnosis (*N* = 1). Given the heterogeneity of follow-up available, we grouped intermediate follow-up (1–2 years) and those with long-term follow-up (more than 3 years) (Supplemental Item 3). All three cohorts had baseline data at or before the time of AKI. There was no difference in baseline length z-scores (mean difference 0.16 [95% CI −0.04, 0.36] *p* = 0.13) nor difference in weight z-scores between those with AKI and those without (mean difference 0.08 [95% CI-0.13, 0.28] *p* = 0.46).

Children with cardiac disease and AKI did not have a difference in growth at 1–2 years after AKI episode in length z-score (mean difference 0.20 [95% CI −0.07, 0.47] *p* = 0.14) nor weight z-score (mean difference 0.13 [95% CI −0.06, 0.32] *p* = 0.19). After 3 years, those with AKI did not have a difference in length z-score (mean difference −0.19 [95% CI −0.53, 0.15] *p* = 0.28) nor weight z-score (mean difference −0.23 [95% CI −0.57, 0.11] *p* = 0.19) compared to those without AKI (Figs. 2 and 3B). We note that in the cardiac cohort, both children with and without AKI had poor growth.

### Non-kidney solid organ transplant cohorts

We included 6 cohorts of children with non-kidney solid organ transplantation (SOT) in this cohort (Table 1), with a total of 1,018 children with SOT and growth parameter follow-up ranging from 12 to 36 months. This included existing cohorts of children with heart (*N* = 4), non-kidney solid organ (*N* = 1) and liver (*N* = 1) transplants (Supplemental Item 4). 5 cohorts had baseline data available. Those with AKI had lower z-scores for length at baseline compared to those without AKI (mean z-score difference of −0.40 [95% CI −0.54, −0.25] *p* < 0.001). Those with AKI had lower z-scores for weight at baseline compared to those without AKI as well (mean z-score difference of −0.23 [95% CI −0.44, −0.02] *p* = 0.04).

Six months following AKI, children with SOT and AKI continued to have lower height than those without AKI (mean z-scores difference of −0.54 [95% CI −0.94, −0.14] *p* = 0.01). This height difference persisted at 12 months (mean z-scores difference of −0.41 [95% CI −0.56, −0.26] *p* < 0.001), 24 months (mean z-scores difference of −0.45 [95% CI −0.60, −0.31] *p* < 0.001), and 36 months (mean z-scores difference of −0.52 [95% CI −0.69, −0.36] *p* < 0.001).

After AKI there was an observed difference in weight in the 2 studies with data at 6 months (mean z-score difference of −0.51 [95% CI −0.91, −0.11] *p* = 0.01). There was no longer a difference at 12 months (mean z-score difference of −0.06 [95% CI −0.40, 0.28] *p* = 0.72) nor at 24 months (mean z-score difference of −0.10 [95% CI −0.68, 0.48] *p* = 0.75). However, at 36 months post-AKI, this difference was again seen with those with AKI having lower weight z-score (mean z-score difference of −0.47 [95% CI −0.76, −0.19] *p* < 0.001) (Figs. 2 and 3C).

### Critically ill children cohorts

We included two cohorts of critically ill children in this meta-analysis (Table 1) with a total of 435 children with follow-up ranging from 3–11 years after AKI. This included existing cohorts of critically ill children without cardiac disease (*N* = 1) and children receiving chemotherapeutic treatment with cisplatin [29, 30] (*N* = 1). Given the heterogeneity of follow-up available, we grouped intermediate follow-up (3 months to 3 years after AKI) and those with long-term follow-up (≥ 3 years) (Supplemental Item 5). Both cohorts had baseline data available. Those with AKI had lower z-scores for length at baseline compared to those without AKI (mean z-score difference of −0.26 [95% CI −0.50, −0.01] *p* = 0.04). There were no differences in weight z-scores at baseline in those with AKI compared to those without AKI as well (mean z-score difference of –0.08 [95% CI −0.28, 0.13] *p* = 0.45).

At 3–36 months after AKI in children with critical illness, those with AKI had lower z-score length compared to those without AKI (mean z-score difference of −0.37 [95% CI −0.57, −0.18] *p* < 0.001). This difference in length persisted longer-term (6 years to 11 years after AKI) with a mean difference in z-score for length of −0.34 (95% CI −0.57, −0.12, *p* < 0.001). There was no difference in weight z-score 3–36 months (*p* = 0.05) nor 6–11 years (*p* = 0.52) after AKI (Figs. 2 and 3D).

## Discussion

In this meta-analysis of observational cohorts of childhood AKI, we report that children and infants with AKI have impaired growth following an episode of AKI. However, these findings varied in effect and persistence during follow-up and depended on the patient population.

Growth impairment during early childhood was seen in infants with an episode of AKI during their admission following birth. While our study was not able to evaluate the causality of this relationship the associative finding may represent the importance of early systemic perturbations in the early growth trajectory of children. This may be particularly notable in small children and infants, both in the short and long term. For infants, half of adult height is achieved by two years of age, and during this phase, an infant may experience a loss in growth potential that cannot be recovered [35, 36]. Except for during the gestational period, linear growth is highest in the first year of life and depends primarily on the provision of optimal nutrition [37].

Our findings are concordant with existing translational and pre-clinical models of growth impacts of AKI which motivated this meta-analysis. In young adult mice with AKI, after initial recovery from an ischemic AKI episode, growth was affected long-term compared to those without AKI. In this study, both male and female mice in the AKI cohort weighed significantly less than healthy and sham controls at 1 year. While the study did not utilize metabolic cages, there were no notable differences in diet or activity level that would otherwise impact these findings. Additionally, there was no apparent reduction in muscle mass, indicating a potential decrease in fat and/or bone [14]. Our preclinical model utilizing rat pups also demonstrated growth differences after AKI compared to sham and illustrated that AKI results in decreased pulmonary vascular growth and alveolarization, with a lung phenotype that mimics bronchopulmonary dysplasia (BPD) [15]. Given that infants with BPD have slower growth velocities, this could further explain why there were growth differences seen in this meta-analysis. While the mechanism of this relationship remains unclear and warrants further study, AKI has been shown to alter the metabolome with alterations in energy utilization and oxidative stress, and these metabolic perturbations may contribute to alterations in growth [38]. Studies to understand the impact of AKI on diet, nutrition, and appetite post-AKI in either humans or pre-clinical models would help to delineate these relationships further [7].

We note that in the cardiac cohort, while no differences between those with and without AKI at baseline nor at follow-up after AKI episode was observed, both groups had very poor growth. Additionally, in children in the cardiac cohort and those undergoing non-kidney solid organ transplants, there were pre-AKI differences in growth, which persisted after an episode of AKI. Impaired growth is a challenge in children with congenital heart disease and solid organ transplants [39, 40]. The reasons for this are likely multifactorial. These patients are often at risk of repeated episodes of AKI, and it is possible that such previous episodes may predispose them to poor growth or represent a bidirectional interplay between AKI and nutrition and growth [26, 41]. Furthermore, there could have been differences in baseline characteristics, treatment course or nutritional exposures which may have further predisposed children to future episodes of AKI [42]. There are gaps in our knowledge about nutrition before, during, and after AKI and children with AKI often receive less nutrition during an episode of AKI [43]. Furthermore, there are no standardized approaches to nutritional assessment after AKI [7]. Further studies are needed to investigate the timing of AKI and growth parameters. Furthermore, monitoring after AKI may optimize the rehabilitative process, allowing for improvements in functional status, lean mass and muscle strength [7].

Despite the strengths and novelty of our analysis, we recognize significant limitations in our data, which limit the generalizability of our findings. First, this analysis included only a convenience sample of observational cohorts of children with AKI that were not designed to assess growth outcomes, and this data was obtained retrospectively. Second, we recognize that our study selection could have been biased, have high heterogeneity, and be non-representative of the generalized population of children with AKI [44]. Third, the differences we observed in long-term growth parameters may in part reflect differences between groups with and without AKI (e.g., developmental stage and expected growth, comorbidities, chronic disease, recurring health problems occurring after the index admission, or neonatal events) or persistence of acute kidney disease (AKD). Finally, we did not evaluate the relationship between persistently decreased kidney function (e.g., AKD or chronic kidney disease) after AKI in these cohorts and its relationship to poor growth.

## Conclusions

In this meta-analysis of observational cohorts of childhood AKI, we found that children and infants with AKI have impaired growth after an episode of AKI. These findings were most striking in infants and differences in growth were of varying effect and persistence during follow-up, depending on the patient population. Our findings suggest a relationship between AKI and poor growth. We suggest clinicians and researchers focus on growth outcomes in both clinical care and research investigating the systemic impacts of AKI episodes. Information about growth after AKI (including weight, height, and ideally head circumference in children < 2 years) should be part of post-AKI follow-up care, such as that performed by post-ICU or post-AKI clinics [45]. Future work should focus on risk factors in children with AKI that predispose to alterations in growth and to identify nutritional targets and interventions to mitigate these serious long-term poor outcomes.

## Supplementary Information

Below is the link to the electronic supplementary material.

Graphical abstract (PPTX 183 KB)

Supplementary file1 (PDF 1.49 MB)

## Data Availability

Group summary data are available from the corresponding author upon reasonable request.
