# Supplementary material for: Growth after pediatric and neonatal acute kidney injury: a meta-analysis
Source: Pediatr Nephrol. 2025 May 9;40(11):3379–89. doi: 10.1007/s00467-025-06801-6 (PMC12484312; doi:10.1007/s00467-025-06801-6)
Supplement: Supplementary file 1 — Graphical abstract (PPTX 183 KB) [file 467_2025_6801_MOESM1_ESM.pptx]

## Slide 1
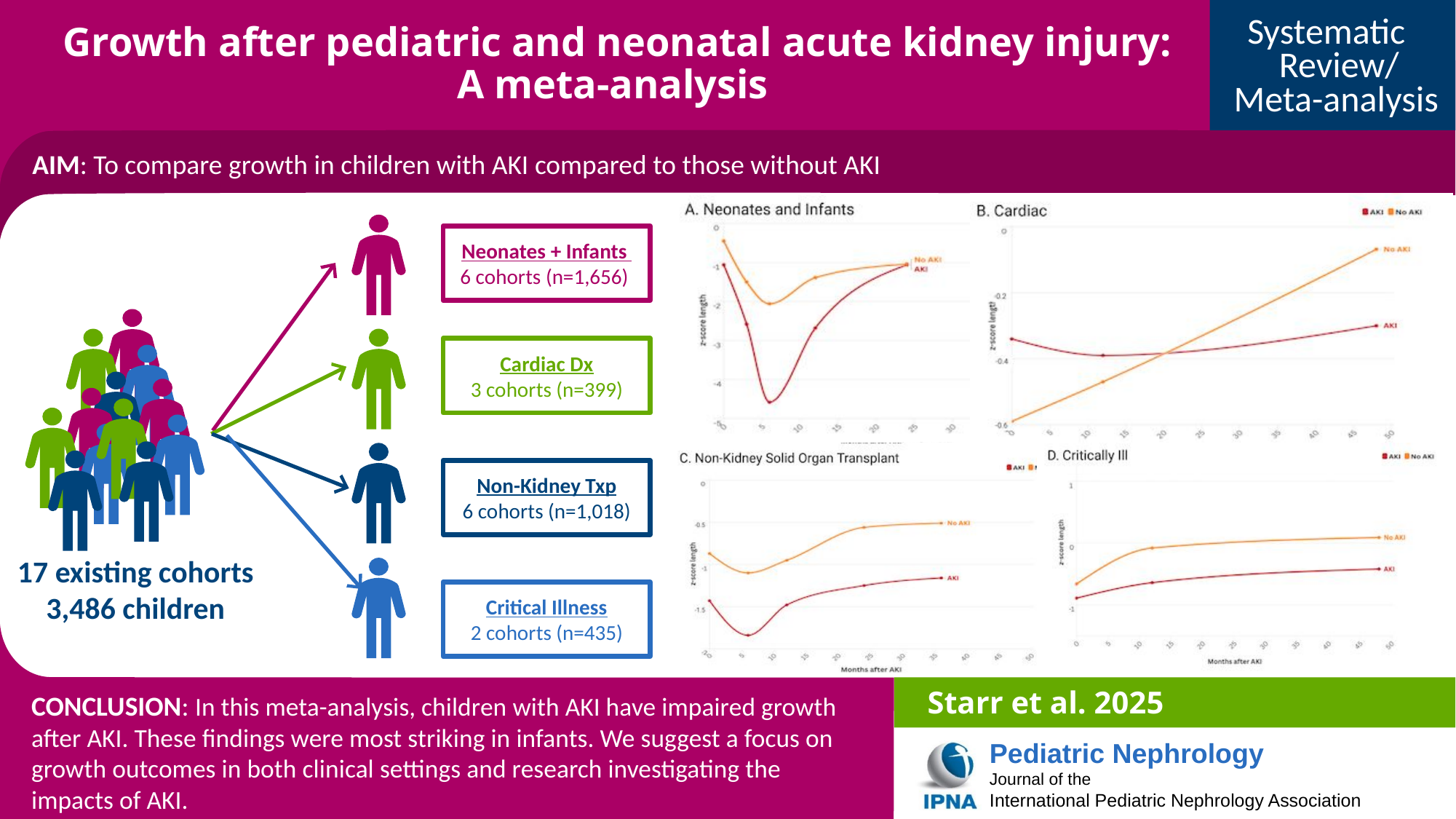

Growth after pediatric and neonatal acute kidney injury:
A meta-analysis
AIM: To compare growth in children with AKI compared to those without AKI
Neonates + Infants
6 cohorts (n=1,656)
Cardiac Dx
3 cohorts (n=399)
Non-Kidney Txp
6 cohorts (n=1,018)
17 existing cohorts
3,486 children
Critical Illness
2 cohorts (n=435)
Starr et al. 2025
CONCLUSION: In this meta-analysis, children with AKI have impaired growth after AKI. These findings were most striking in infants. We suggest a focus on growth outcomes in both clinical settings and research investigating the impacts of AKI.
