# Supplementary material for: Growth after pediatric and neonatal acute kidney injury: a meta-analysis
Source: Pediatr Nephrol. 2025 May 9;40(11):3379–89. doi: 10.1007/s00467-025-06801-6 (PMC12484312; doi:10.1007/s00467-025-06801-6)

Growth After Pediatric and Neonatal Acute Kidney Injury: A Meta-Analysis

Michelle C. Starr MD MPH @mcstarr @mcstarr1.bsky.social 1[1,2], Mital Patel MD[3,4], Faizeen Zafar MD[5,6], Melissa S. Zhou MD @MelissaSZhou[7], Russell Griffin Ph.D[8], Annabel Biruete[,10], Vedran Cockovski PhD[5], Rasheed Gbadegesin MD[4], Dana Y. Fuhrman DO MS[11], Katja M. Gist DO[12], Cherry Mammen MD[13], Shina Menon MD[7], Catherine Morgan MD[14], Cara L. Slagle MD MS[15], Scott Sutherland MD[7], Michael Zappitelli MD[5], Danielle E. Soranno MD[1,16]

Supplemental Items

Supplemental Item 1: MOOSE

Supplemental Item 2: Forest plot of standardized mean differences and 95% CIs for growth z-scores for 6 included studies of neonates and infants showing length-for-age and weight-for-age z-scores at baseline and 2-3 months, 6 months, 12 months and 24 months after AKI.

Supplemental Item 3: Forest plot of standardized mean differences and 95% CIs for growth z-scores for 3 included studies of children with cardiac disease showing length-for-age and weight-for-age z-scores at baseline, 1-2 years after AKI and more than 3 years after AKI.

Supplemental Item 4: Forest plot of standardized mean differences and 95% CIs for growth z-scores for 6 included studies of children with non-kidney solid organ transplantation showing length-for-age and weight-for-age z-scores at baseline as well as 6 months, 12 months, 24 months and 36 months after AKI.

Supplemental Item 5: Forest plot of standardized mean differences and 95% CIs for growth z-scores for 2 included studies of children with non-cardiac critical illness showing length-for-age and weight-for-age z-scores at baseline as well as 3 months to less than 3 years after AKI. and more than 3 years after AKI.

Supplemental Item 1

MOOSE (Meta-analyses Of Observational Studies in Epidemiology) Checklist

A reporting checklist for Authors, Editors, and Reviewers of Meta-analyses of Observational Studies. You must report the page number in your manuscript where you consider each of the items listed in this checklist. If you have not included this information, either revise your manuscript accordingly before submitting or note N/A.

Reporting Criteria	Reported (Yes/No)	Reported on Page No.
**Reporting of Background**		
Problem definition	Yes	4
Hypothesis statement	Yes	4
Description of Study Outcome(s)	Yes	5
Type of exposure or intervention used	Yes	5
Type of study design used	Yes	5, 6
Study population	Yes	5
**Reporting of Search Strategy**		
Qualifications of searchers (eg, librarians and investigators)	Yes	5
Search strategy, including time period included in the synthesis and keywords	No	NA
Effort to include all available studies, including contact with authors	No	NA
Databases and registries searched	No	NA
Search software used, name and version, including special features used (eg, explosion)	No	NA
Use of hand searching (eg, reference lists of obtained articles)	No	NA
List of citations located and those excluded, including justification	No	NA
Method for addressing articles published in languages other than English	No	NA
Method of handling abstracts and unpublished studies	No	NA
Description of any contact with authors	No	NA
**Reporting of Methods**		
Description of relevance or appropriateness of studies assembled for assessing the hypothesis to be tested	Yes	6
Rationale for the selection and coding of data (eg, sound clinical principles or convenience)	Yes	6
Documentation of how data were classified and coded (eg, multiple raters, blinding, and interrater reliability)	Yes	6
Assessment of confounding (eg, comparability of cases and controls in studies where appropriate	Yes	6

Reporting Criteria	Reported (Yes/No)	Reported on Page No.
Assessment of study quality, including blinding of quality assessors; stratification or regression on possible predictors of study results	No	NA
Assessment of heterogeneity	Yes	6
Description of statistical methods (eg, complete description of fixed or random effects models, justification of whether the chosen models account for predictors of study results, dose-response models, or cumulative meta-analysis) in sufficient detail to be replicated	Yes	6
Provision of appropriate tables and graphics	Yes	6, Supp Items
**Reporting of Results**		
Table giving descriptive information for each study included	Yes	Table 1
Results of sensitivity testing (eg, subgroup analysis)	Yes	8-11
Indication of statistical uncertainty of findings	Yes	8-11
**Reporting of Discussion**		
Quantitative assessment of bias (eg, publication bias)	No	NA
Justification for exclusion (eg, exclusion of non–English-language citations)	No	NA
Assessment of quality of included studies	Yes	8
**Reporting of Conclusions**		
Consideration of alternative explanations for observed results	Yes	12, 13
Generalization of the conclusions (ie, appropriate for the data presented and within the domain of the literature review)	Yes	13, 14
Guidelines for future research	Yes	13, 14
Disclosure of funding source	Yes	1

Once you have completed this checklist, please save a copy and upload it as part of your submission. DO NOT include this checklist as part of the main manuscript document. It must be uploaded as a separate file.

Supplemental Item 2

Length

Baseline

Study	AKI N	AKI Mean	AKI SD	No AKI N	No AKI Mean	No AKI SD	Hedges's g with 95% CI	Weight (%)
Patel	219	-0.68	1.32	26	-0.91	1.67	0.17 [-0.24, 0.58]	24.14
Patel	57	-0.9	1.22	222	-0.27	1.39	-0.46 [-0.76, -0.17]	25.34
Starr	107	-1.91	1.94	99	-1.32	1.67	-0.32 [-0.60, -0.05]	25.51
Slagle	56	-1.113	.493	173	-.119	.763	-1.40 [-1.73, -1.07]	25.01
**Overall**							-0.51 [-1.15, 0.13]	

Heterogeneity: $\tau^2 = 0.39$, $I^2 = 93.66\%$, $H^2 = 15.78$

Test of $\theta_i = \theta_j$: $Q(3) = 40.77$, $p = 0.00$

Test of $\theta = 0$: $z = -1.57$, $p = 0.12$

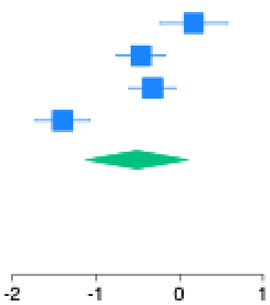

2-3 Months

Study	AKI N	AKI Mean	AKI SD	No AKI N	No AKI Mean	No AKI SD	Hedges's g with 95% CI	Weight (%)
Patel	219	-2.21	1.84	26	-1.71	1.44	-0.28 [-0.68, 0.13]	15.31
Patel	57	-1.93	1.96	222	-0.60	1.84	-0.71 [-1.01, -0.42]	24.95
Starr	107	-2.72	1.73	12	-1.97	1.72	-0.43 [-1.03, 0.16]	7.85
Starr	107	-3.74	1.81	99	-2.97	1.57	-0.45 [-0.73, -0.18]	27.58
Slagle	56	-2.19	1.69	173	-1.74	1.40	-0.31 [-0.61, -0.01]	24.30
**Overall**							-0.45 [-0.63, -0.28]	

Heterogeneity: $\tau^2 = 0.01$, $I^2 = 22.77\%$, $H^2 = 1.29$

Test of $\theta_i = \theta_j$: $Q(4) = 4.55$, $p = 0.34$

Test of $\theta = 0$: $z = -5.07$, $p = 0.00$

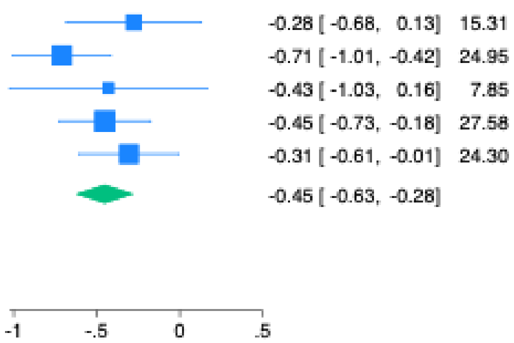

6 Months

Study	N	Mean	SD	N	Mean	SD	with 95% CI	(%)
Patel	219	-5.5	3.81	26	-3.56	1.57	-0.53 [-0.94, -0.12]	18.69
Patel	57	-3.23	1.7	222	-1.61	1.38	-1.11 [-1.42, -0.81]	23.27
Starr	107	-3.72	2.7	10	-2.01	1.29	-0.65 [-1.30, 0.00]	11.07
Starr	107	-4.67	2.81	99	-3.01	1.73	-0.70 [-0.98, -0.42]	24.39
Slagle	56	-3.875	1.813	173	-1.87	1.65	-1.18 [-1.50, -0.86]	22.57
**Overall**							-0.87 [-1.13, -0.60]	

Heterogeneity: $\tau^2 = 0.05$, $I^2 = 62.27\%$, $H^2 = 2.65$

Test of $\theta_i = \theta_j$: $Q(4) = 10.42$, $p = 0.03$

Test of $\theta = 0$: $z = -6.41$, $p = 0.00$

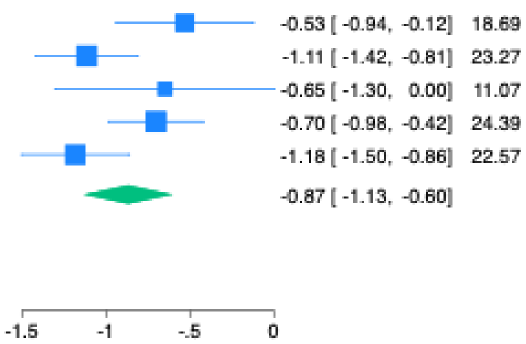

12 Months

Study	AKI N	AKI Mean	AKI SD	No AKI N	No AKI Mean	No AKI SD	Hedges's g with 95% CI	Weight (%)
Patel	219	-3.15	2.6	26	-2.7	1.38	-0.18 [-0.59, 0.23]	19.99
Patel	57	-2.27	1.65	222	-1.21	2.83	-0.40 [-0.69, -0.11]	22.68
Starr	107	-2.17	1.7	7	-1.92	1.94	-0.14 [-0.91, 0.62]	12.32
Starr	107	-2.35	1.43	99	-1.38	1.4	-0.68 [-0.96, -0.40]	22.94
Slagle	56	-2.865	0.8	173	-1.415	1.365	-1.15 [-1.47, -0.83]	22.07
**Overall**							-0.56 [-0.92, -0.19]	

Heterogeneity: $\tau^2 = 0.13$, $I^2 = 80.01\%$, $H^2 = 5.00$

Test of $\theta_i = \theta_j$: $Q(4) = 19.06$, $p = 0.00$

Test of $\theta = 0$: $z = -2.97$, $p = 0.00$

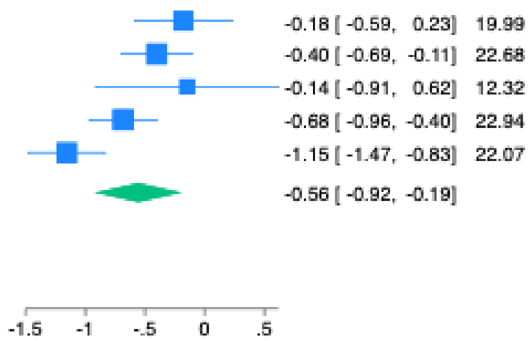

24 Months

Study	AKI N	AKI Mean	AKI SD	No AKI N	No AKI Mean	No AKI SD	Hedges's g with 95% CI	Weight (%)
Starr	155	-1.16	1.15	542	-1.04	1.53	-0.08 [-0.26, 0.10]	96.90
Starr	107	-0.91	1.59	4	-0.51	2.1	-0.25 [-1.25, 0.75]	3.10
**Overall**							-0.09 [-0.26, 0.09]	

Heterogeneity: $\tau^2 = 0.00$, $I^2 = 0.00\%$, $H^2 = 1.00$

Test of $\theta_i = \theta_j$: $Q(1) = 0.10$, $p = 0.75$

Test of $\theta = 0$: $z = -0.98$, $p = 0.33$

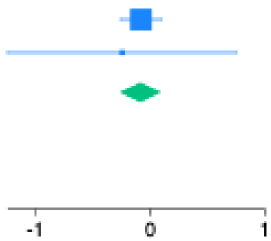

Weight

Baseline

Study	AKI N	AKI Mean	AKI SD	No AKI N	No AKI Mean	No AKI SD	Hedges's g with 95% CI	Weight (%)
Patel	219	-0.65	1.1	26	-1.57	2.65	0.68 [0.27, 1.09]	24.53
Patel	57	-1.15	1.61	222	-0.4	1.36	-0.53 [-0.82, -0.23]	25.18
Starr	107	-1.87	2.12	99	-2.61	2.17	0.34 [0.07, 0.62]	25.27
Slagle	56	-1.114	1.419	173	-.001	.528	-1.33 [-1.65, -1.00]	25.02
**Overall**							-0.21 [-1.09, 0.67]	

Heterogeneity: $\tau^2 = 0.78$, $I^2 = 96.71\%$, $H^2 = 30.39$

Test of $\theta_i = \theta_j$: $Q(3) = 83.15$, $p = 0.00$

Test of $\theta = 0$: $z = -0.47$, $p = 0.64$

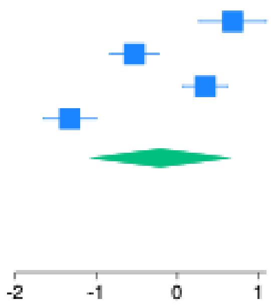

2-3 Months

Study	Treatment N	Treatment Mean	Treatment SD	Control N	Control Mean	Control SD	Hedges's g with 95% CI	Weight (%)
Patel	219	-1.70	1.57	26	-0.45	1.23	-0.81 [-1.22, -0.40]	16.89
Patel	57	-1.77	1.74	222	-0.60	1.84	-0.64 [-0.94, -0.35]	24.05
Starr	107	-2.15	1.83	12	-1.42	1.30	-0.41 [-1.00, 0.19]	10.13
Starr	107	-2.98	1.58	99	-2.01	1.64	-0.60 [-0.88, -0.32]	25.30
Slagle	56	-1.44	1.57	173	-1.17	1.25	-0.20 [-0.50, 0.10]	23.63
**Overall**							-0.53 [-0.75, -0.32]	

Heterogeneity: $\tau^2 = 0.03$, $I^2 = 47.43\%$, $H^2 = 1.90$

Test of $\theta_i = \theta_j$: $Q(4) = 7.37$, $p = 0.12$

Test of $\theta = 0$: $z = -4.82$, $p = 0.00$

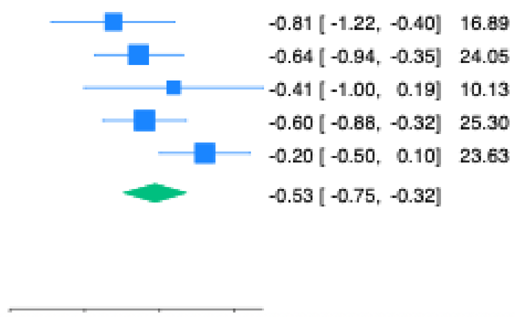

6 Months

Study	AKI N	AKI Mean	AKI SD	No AKI N	No AKI Mean	No AKI SD	Hedges's g with 95% CI	Weight (%)
Patel	219	-3.81	4.65	26	-2.87	1.13	-0.21 [-0.62, 0.19]	19.16
Patel	57	-2.56	1.44	222	-0.9	3.93	-0.46 [-0.76, -0.17]	22.97
Starr	107	-3.14	1.74	10	-2.34	1.01	-0.47 [-1.12, 0.18]	12.46
Starr	107	-3.01	1.62	99	-1.5	1.37	-1.00 [-1.29, -0.71]	23.08
Slagle	56	-2.31	1.145	173	-1.3	1.12	-0.89 [-1.21, -0.58]	22.33
**Overall**							-0.64 [-0.94, -0.33]	

Heterogeneity: $\tau^2 = 0.08$, $I^2 = 71.59\%$, $H^2 = 3.52$

Test of $\theta_i = \theta_j$: $Q(4) = 14.02$, $p = 0.01$

Test of $\theta = 0$: $z = -4.11$, $p = 0.00$

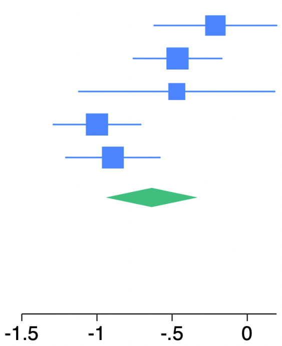

12 Months

Study	AKI N	AKI Mean	AKI SD	No AKI N	No AKI Mean	No AKI SD	Hedges's g with 95% CI	Weight (%)
Patel	219	-1.95	1.45	26	-1.69	1.06	-0.18 [-0.59, 0.22]	18.77
Patel	57	-1.4	1.47	222	-0.33	1.18	-0.86 [-1.16, -0.56]	23.85
Starr	107	-1.31	1.41	7	-0.99	1.23	-0.23 [-0.99, 0.54]	8.61
Starr	107	-2.01	1.4	99	-1.47	1.32	-0.39 [-0.67, -0.12]	25.07
Slagle	56	-0.985	1.28	173	-0.68	0.82	-0.32 [-0.62, -0.02]	23.69
**Overall**							-0.43 [-0.69, -0.17]	

Heterogeneity: $\tau^2 = 0.05$, $I^2 = 60.59\%$, $H^2 = 2.54$

Test of $\theta_i = \theta_j$: $Q(4) = 9.94$, $p = 0.04$

Test of $\theta = 0$: $z = -3.28$, $p = 0.00$

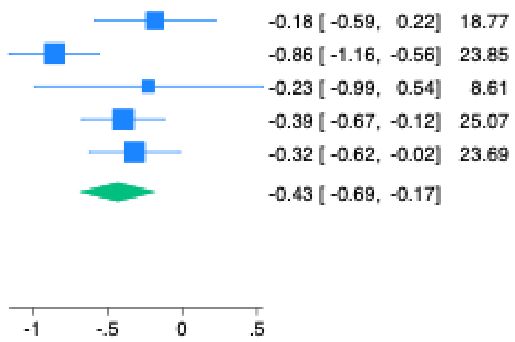

24 Months

Study	AKI N	AKI Mean	AKI SD	No AKI N	No AKI Mean	No AKI SD	Hedges's g with 95% CI	Weight (%)
Starr	155	-1.19	1.35	542	-1.11	1.46	-0.06 [-0.23, 0.12]	96.90
Starr	107	-0.56	1.53	4	-0.2	1.3	-0.23 [-1.23, 0.76]	3.10
**Overall**							-0.06 [-0.24, 0.11]	

Heterogeneity: $\tau^2 = 0.00$, $I^2 = 0.00\%$, $H^2 = 1.00$

Test of $\theta_i = \theta_j$: $Q(1) = 0.12$, $p = 0.73$

Test of $\theta = 0$: $z = -0.68$, $p = 0.50$

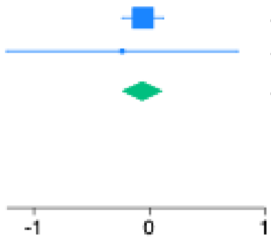

Supplemental Item 3

Baseline

Length

Study	AKI N	AKI Mean	AKI SD	No AKI N	No AKI Mean	No AKI SD	Hedges's g with 95% CI	Weight (%)
Morgan	97	-0.31	1.43	140	-0.62	1.40	0.22 [-0.04, 0.48]	60.44
Zappitelli	22	-1.06	1.98	29	-0.62	1.74	-0.23 [-0.79, 0.32]	13.18
Gist	73	-0.17	1.28	38	-0.43	1.10	0.21 [-0.18, 0.60]	26.38
**Overall**							0.16 [-0.04, 0.36]	

Heterogeneity: $\tau^2 = 0.00$, $I^2 = 0.00\%$, $H^2 = 1.00$

Test of $\theta_i = \theta_j$: $Q(2) = 2.20$, $p = 0.33$

Test of $\theta = 0$: $z = 1.52$, $p = 0.13$

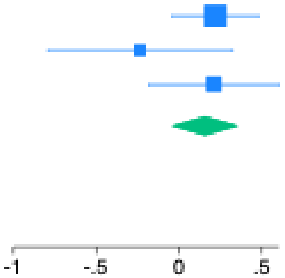

Weight

Study	AKI N	AKI Mean	AKI SD	No AKI N	No AKI Mean	No AKI SD	Hedges's g with 95% CI	Weight (%)
Morgan	97	-0.67	1.26	140	-0.72	1.2	0.04 [-0.22, 0.30]	60.50
Zappitelli	22	1.00	8.51	29	-0.33	1.6	0.23 [-0.33, 0.79]	13.12
Gist	73	-0.59	1.11	38	-0.68	1.17	0.08 [-0.31, 0.47]	26.38
**Overall**							0.08 [-0.13, 0.28]	

Heterogeneity: $\tau^2 = 0.00$, $I^2 = 0.00\%$, $H^2 = 1.00$

Test of $\theta_i = \theta_j$: $Q(2) = 0.37$, $p = 0.83$

Test of $\theta = 0$: $z = 0.74$, $p = 0.46$

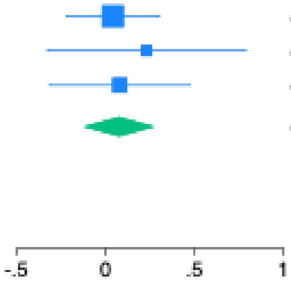

1-2 Years

Length

Study	AKI N	AKI Mean	AKI SD	No AKI N	No AKI Mean	No AKI SD	Hedges's g with 95% CI	Weight (%)
Morgan	97	-0.24	1.21	140	-0.24	0.97	0.00 [-0.26, 0.26]	44.24
Zappitelli	69	-0.48	1.16	37	-0.96	1.19	0.41 [0.00, 0.81]	27.91
Gist	67	-0.51	1.00	37	-0.84	1.12	0.31 [-0.09, 0.72]	27.86
**Overall**							0.20 [-0.07, 0.47]	

Heterogeneity: $\tau^2 = 0.03$, $I^2 = 43.88\%$, $H^2 = 1.78$

Test of $\theta_i = \theta_j$: $Q(2) = 3.47$, $p = 0.18$

Test of $\theta = 0$: $z = 1.47$, $p = 0.14$

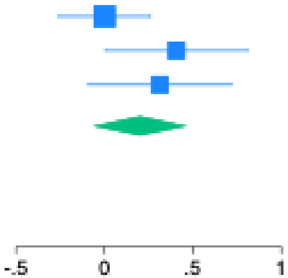

Weight

Study	AKI N	AKI Mean	AKI SD	No AKI N	No AKI Mean	No AKI SD	Hedges's g with 95% CI	Weight (%)
Morgan	97	-0.33	1.21	140	-0.44	1.39	0.08 [-0.18, 0.34]	54.57
Zappitelli	69	-1.06	1.28	37	-1.44	1.34	0.29 [-0.11, 0.69]	22.73
Gist	67	-0.51	1.24	37	-0.60	1.31	0.07 [-0.33, 0.47]	22.70
**Overall**							0.13 [-0.06, 0.32]	

Heterogeneity: $\tau^2 = 0.00$, $I^2 = 0.00\%$, $H^2 = 1.00$

Test of $\theta_i = \theta_j$: $Q(2) = 0.82$, $p = 0.66$

Test of $\theta = 0$: $z = 1.30$, $p = 0.19$

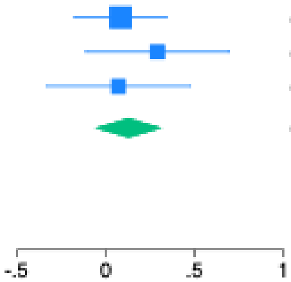

>3 Years

Length

Study	AKI N	AKI Mean	AKI SD	No AKI N	No AKI Mean	No AKI SD	Hedges's g with 95% CI	Weight (%)
Zappitelli	22	-0.29	1.08	29	0.01	0.99	-0.29 [-0.84, 0.27]	37.63
Gist	61	-0.30	1.16	31	-0.14	1.30	-0.13 [-0.56, 0.30]	62.37
**Overall**							-0.19 [-0.53, 0.15]	

Heterogeneity: $\tau^2 = 0.00$, $I^2 = 0.00\%$, $H^2 = 1.00$

Test of $\theta_i = \theta_j$: $Q(1) = 0.19$, $p = 0.67$

Test of $\theta = 0$: $z = -1.09$, $p = 0.28$

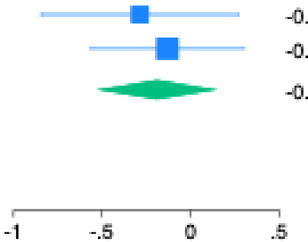

Weight

Study	AKI N	AKI Mean	AKI SD	No AKI N	No AKI Mean	No AKI SD	Hedges's g with 95% CI	Weight (%)
Zappitelli	22	-0.16	1.51	29	0.15	0.95	-0.25 [-0.81, 0.31]	37.77
Gist	61	-0.84	1.22	31	-0.57	1.23	-0.22 [-0.65, 0.21]	62.23
**Overall**							-0.23 [-0.57, 0.11]	

Heterogeneity: $\tau^2 = 0.00$, $I^2 = 0.00\%$, $H^2 = 1.00$

Test of $\theta_i = \theta_j$: $Q(1) = 0.01$, $p = 0.93$

Test of $\theta = 0$: $z = -1.32$, $p = 0.19$

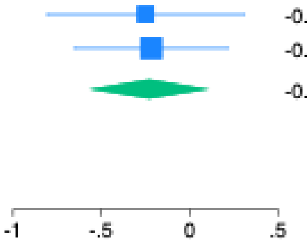

Supplemental Item 4

Length

Baseline

Study	Treatment N	Treatment Mean	Treatment SD	Control N	Control Mean	Control SD	Hedges's g with 95% CI	Weight (%)
Menon	31	-1.89	1.68	36	-1.42	1.80	-0.27 [-0.74, 0.21]	9.66
Menon	14	-2.09	1.50	62	-1.30	1.75	-0.46 [-1.04, 0.12]	6.56
Fuhrman	186	-1.40	1.26	152	-0.67	1.66	-0.50 [-0.72, -0.28]	46.58
Zafar	27	-0.89	1.58	74	-0.76	1.60	-0.08 [-0.52, 0.36]	11.48
Zhou	126	-1.40	1.68	71	-0.77	1.58	-0.38 [-0.67, -0.09]	25.73
**Overall**							-0.40 [-0.54, -0.25]	

Heterogeneity: $\tau^2 = 0.00$, $I^2 = 0.00\%$, $H^2 = 1.00$

Test of $\theta_i = \theta_j$: $Q(4) = 3.23$, $p = 0.52$

Test of $\theta = 0$: $z = -5.25$, $p = 0.00$

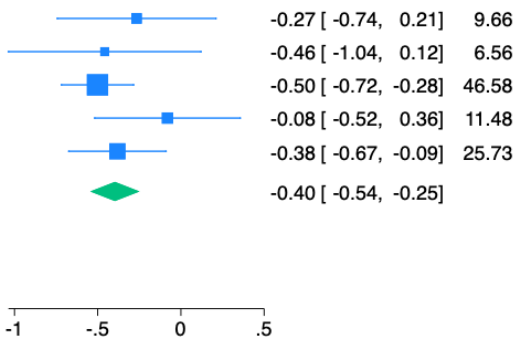

6 Months

Study	AKI N	AKI Mean	AKI SD	No AKI N	No AKI Mean	No AKI SD	Hedges's g with 95% CI	Weight (%)
Menon	28	-1.81	1.22	35	-0.92	1.81	-0.56 [-1.06, -0.05]	61.93
Menon	11	-1.90	1.44	62	-1.20	1.37	-0.50 [-1.15, 0.14]	38.07
**Overall**							-0.54 [-0.94, -0.14]	

Heterogeneity: $\tau^2 = 0.00$, $I^2 = 0.00\%$, $H^2 = 1.00$

Test of $\theta_i = \theta_j$: $Q(1) = 0.02$, $p = 0.89$

Test of $\theta = 0$: $z = -2.63$, $p = 0.01$

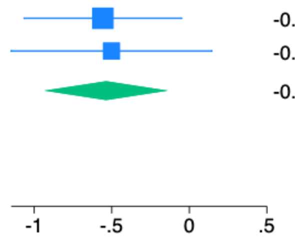

12 Months

Study	AKI N	AKI Mean	AKI SD	No AKI N	No AKI Mean	No AKI SD	Hedges's g with 95% CI	Weight (%)
Menon	27	-1.85	1.01	30	-1.03	1.55	-0.61 [-1.14, -0.08]	8.03
Menon	11	-1.61	1.44	61	-1.13	1.32	-0.35 [-1.00, 0.29]	5.49
Fuhrman	186	-1.35	1.34	152	-0.84	1.10	-0.41 [-0.63, -0.19]	48.63
Zafar	27	-1.27	0.43	74	-1.03	1.63	-0.17 [-0.61, 0.27]	11.71
Zhou	124	-1.62	1.50	71	-0.91	1.56	-0.46 [-0.76, -0.17]	26.14
**Overall**							-0.41 [-0.56, -0.26]	

Heterogeneity: $\tau^2 = 0.00$, $I^2 = 0.00\%$, $H^2 = 1.00$

Test of $\theta_i = \theta_j$: $Q(4) = 1.86$, $p = 0.76$

Test of $\theta = 0$: $z = -5.32$, $p = 0.00$

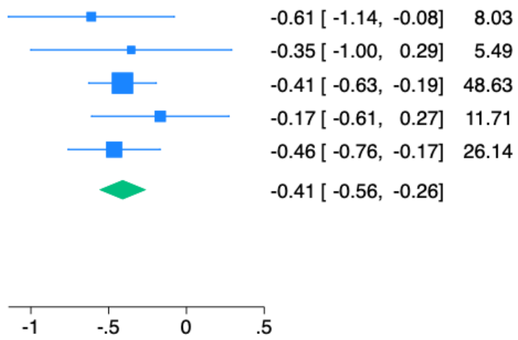

24 Months

Study	AKI N	AKI Mean	AKI SD	No AKI N	No AKI Mean	No AKI SD	Hedges's g with 95% CI	Weight (%)
Menon	25	-1.91	0.97	28	-1.02	1.47	-0.70 [-1.25, -0.14]	6.49
Menon	8	-1.03	1.23	56	-0.96	1.30	-0.05 [-0.79, 0.69]	3.70
Fuhrman	186	-1.33	1.30	152	-0.57	1.28	-0.59 [-0.81, -0.37]	37.50
Morgan	66	-0.51	1.23	171	-0.13	1.05	-0.34 [-0.63, -0.06]	23.22
Zafar	24	-1.38	1.32	61	-0.83	1.52	-0.37 [-0.85, 0.10]	8.82
Zhou	116	-1.38	1.45	64	-0.84	1.53	-0.36 [-0.67, -0.06]	20.26
**Overall**							-0.45 [-0.60, -0.31]	

Heterogeneity: $\tau^2 = 0.00$, $I^2 = 5.18\%$, $H^2 = 1.05$

Test of $\theta_i = \theta_j$: $Q(5) = 4.30$, $p = 0.51$

Test of $\theta = 0$: $z = -6.20$, $p = 0.00$

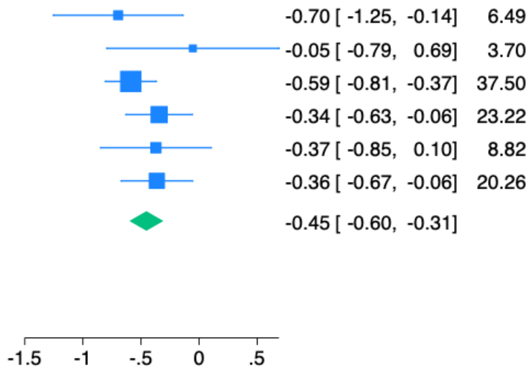

36 Months

Study	AKI N	AKI Mean	AKI SD	No AKI N	No AKI Mean	No AKI SD	Hedges's g with 95% CI	Weight (%)
Menon	21	-1.83	0.96	23	-0.91	1.46	-0.72 [-1.34, -0.11]	7.28
Fuhrman	186	-1.06	0.93	152	-0.30	1.57	-0.60 [-0.82, -0.38]	56.86
Zafar	20	-1.23	1.35	53	-0.74	1.50	-0.33 [-0.85, 0.19]	10.20
Zhou	100	-1.19	1.39	58	-0.67	1.40	-0.37 [-0.70, -0.05]	25.67
**Overall**							-0.52 [-0.69, -0.36]	

Heterogeneity: $\tau^2 = 0.00$, $I^2 = 0.00\%$, $H^2 = 1.00$

Test of $\theta_i = \theta_j$: $Q(3) = 2.28$, $p = 0.52$

Test of $\theta = 0$: $z = -6.22$, $p = 0.00$

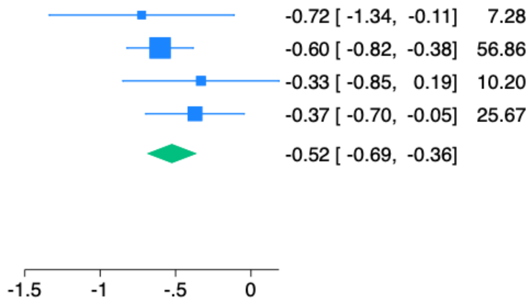

Weight

Baseline

Study	Treatment N	Treatment Mean	Treatment SD	Control N	Control Mean	Control SD	Hedges's g with 95% CI	Weight (%)
Menon	31	-1.47	1.62	36	-1.22	1.30	-0.17 [-0.65, 0.31]	14.40
Menon	14	-0.98	1.35	62	-0.55	1.47	-0.29 [-0.87, 0.28]	10.82
Fuhrman	186	-0.66	1.46	152	-0.13	1.27	-0.38 [-0.60, -0.17]	32.89
Zafar	27	-0.10	1.36	74	-0.46	1.52	0.24 [-0.20, 0.68]	16.10
Zhou	126	-0.78	1.55	71	-0.27	1.45	-0.34 [-0.63, -0.04]	25.79
**Overall**							-0.23 [-0.44, -0.02]	

Heterogeneity: $\tau^2 = 0.02$, $I^2 = 42.41\%$, $H^2 = 1.74$

Test of $\theta_i = \theta_j$: $Q(4) = 6.66$, $p = 0.15$

Test of $\theta = 0$: $z = -2.10$, $p = 0.04$

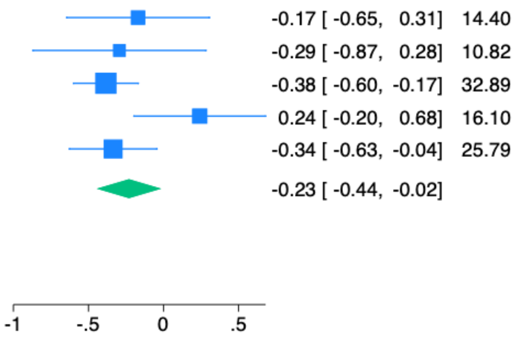

6 Months

Study	AKI N	AKI Mean	AKI SD	No AKI N	No AKI Mean	No AKI SD	Hedges's g with 95% CI	Weight (%)
Menon	28	-1.55	1.06	35	-0.65	1.65	-0.63 [-1.14, -0.12]	61.45
Menon	11	-0.71	0.74	62	-0.35	1.17	-0.32 [-0.96, 0.33]	38.55
**Overall**							-0.51 [-0.91, -0.11]	

Heterogeneity: $\tau^2 = 0.00$, $I^2 = 0.00\%$, $H^2 = 1.00$

Test of $\theta_i = \theta_j$: $Q(1) = 0.54$, $p = 0.46$

Test of $\theta = 0$: $z = -2.49$, $p = 0.01$

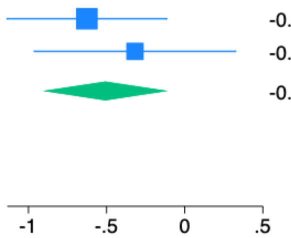

12 Months

Study	Treatment N	Treatment Mean	Treatment SD	Control N	Control Mean	Control SD	Hedges's g with 95% CI	Weight (%)
Menon	27	-0.90	0.76	30	-0.52	1.18	-0.37 [-0.89, 0.14]	17.12
Menon	11	-0.19	0.85	61	0.04	1.05	-0.22 [-0.86, 0.41]	14.19
Fuhrman	186	0.65	1.22	152	0.20	0.89	0.41 [0.20, 0.63]	25.69
Zafar	27	-0.45	1.54	74	-0.53	1.62	0.05 [-0.39, 0.49]	19.36
Zhou	126	-0.59	1.49	71	-0.05	1.64	-0.35 [-0.64, -0.06]	23.64
**Overall**							-0.06 [-0.40, 0.28]	

Heterogeneity: $\tau^2 = 0.10$, $I^2 = 74.85\%$, $H^2 = 3.98$

Test of $\theta_i = \theta_j$: $Q(4) = 21.27$, $p = 0.00$

Test of $\theta = 0$: $z = -0.36$, $p = 0.72$

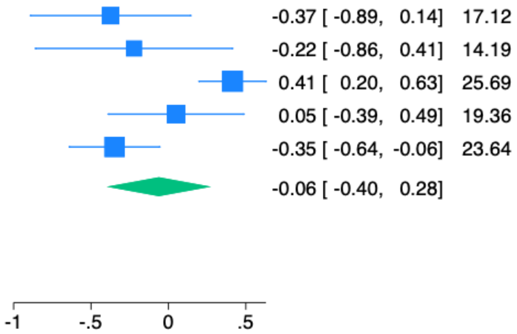

24 Months

Study	AKI N	AKI Mean	AKI SD	No AKI N	No AKI Mean	No AKI SD	Hedges's g with 95% CI	Weight (%)
Menon	25	0.90	0.65	28	-0.28	1.39	1.05 [0.47, 1.63]	15.69
Menon	8	-0.04	0.71	56	0.04	1.05	-0.08 [-0.82, 0.66]	14.27
Fuhrman	186	-0.82	0.84	152	0.18	0.83	-1.19 [-1.43, -0.96]	18.04
Zafar	24	-0.25	1.69	61	-0.44	1.52	0.12 [-0.35, 0.59]	16.54
Zhou	117	-0.38	1.43	65	-0.13	1.96	-0.15 [-0.46, 0.15]	17.68
Morgan	66	-0.51	1.16	171	-0.31	1.31	-0.16 [-0.44, 0.13]	17.78
**Overall**							-0.10 [-0.68, 0.48]	

Heterogeneity: $\tau^2 = 0.47$, $I^2 = 93.31\%$, $H^2 = 14.94$

Test of $\theta_i = \theta_j$: $Q(5) = 79.52$, $p = 0.00$

Test of $\theta = 0$: $z = -0.33$, $p = 0.75$

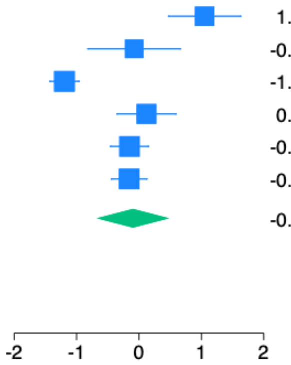

36 Months

Study	Treatment N	Treatment Mean	Treatment SD	Control N	Control Mean	Control SD	Hedges's g with 95% CI	Weight (%)
Menon	21	-0.88	0.76	23	-0.44	1.57	-0.35 [-0.93, 0.24]	15.66
Fuhrman	186	-0.80	1.05	152	-0.04	0.98	-0.74 [-0.97, -0.52]	36.36
Zafar	20	-0.20	1.73	53	-0.04	1.61	-0.10 [-0.61, 0.41]	18.68
Zhou	101	-0.36	1.41	61	0.31	1.67	-0.44 [-0.76, -0.12]	29.30
**Overall**							-0.47 [-0.76, -0.19]	

Heterogeneity: $\tau^2 = 0.05$, $I^2 = 56.50\%$, $H^2 = 2.30$

Test of $\theta_i = \theta_j$: $Q(3) = 6.90$, $p = 0.08$

Test of $\theta = 0$: $z = -3.25$, $p = 0.00$

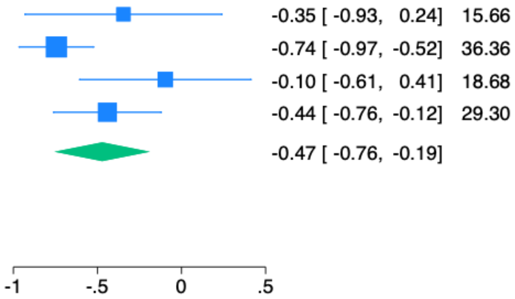

Supplemental Item 5

Length

Baseline

Study	AKI N	AKI Mean	AKI SD	No AKI N	No AKI Mean	No AKI SD	Hedges's g with 95% CI	Weight (%)
Zappitelli	73	-0.45	1.09	86	0.07	1.79	-0.34 [-0.66, -0.03]	61.96
Zappitelli	33	-1.86	4.13	86	-1.39	3.73	-0.12 [-0.52, 0.28]	38.04
**Overall**							-0.26 [-0.50, -0.01]	

Heterogeneity: $\tau^2 = 0.00$, $I^2 = 0.00\%$, $H^2 = 1.00$

Test of $\theta_i = \theta_j$: $Q(1) = 0.73$, $p = 0.39$

Test of $\theta = 0$: $z = -2.06$, $p = 0.04$

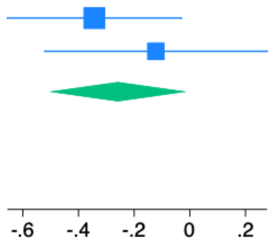

3 Months – <3 Years

Study	AKI N	AKI Mean	AKI SD	No AKI N	No AKI Mean	No AKI SD	Hedges's g with 95% CI	Weight (%)
Zappitelli (3m)	70	-0.58	1.05	85	0.05	1.75	-0.42 [-0.74, -0.11]	37.78
Zappitelli (12m)	64	-0.68	1.09	84	-0.13	1.71	-0.37 [-0.70, -0.04]	35.96
Zappitelli (3y)	48	-0.68	1.69	58	-0.21	1.50	-0.29 [-0.68, 0.09]	26.27
**Overall**							-0.37 [-0.57, -0.18]	

Heterogeneity: $\tau^2 = 0.00$, $I^2 = 0.00\%$, $H^2 = 1.00$

Test of $\theta_i = \theta_j$: $Q(2) = 0.27$, $p = 0.88$

Test of $\theta = 0$: $z = -3.72$, $p = 0.00$

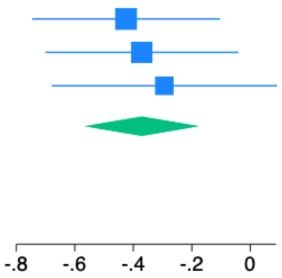

>3 Years

Study	AKI N	AKI Mean	AKI SD	No AKI N	No AKI Mean	No AKI SD	Hedges's g with 95% CI	Weight (%)
Zappitelli (6y)	58	-0.45	1.37	185	0.26	1.45	-0.49 [-0.79, -0.20]	48.15
Zappitelli (7.5y)	41	-0.44	1.47	111	-0.13	1.41	-0.22 [-0.57, 0.14]	35.02
Zappitelli (11y)	18	-0.29	1.20	51	-0.08	1.19	-0.17 [-0.71, 0.36]	16.83
**Overall**							-0.34 [-0.57, -0.12]	

Heterogeneity: $\tau^2 = 0.00$, $I^2 = 10.05\%$, $H^2 = 1.11$

Test of $\theta_i = \theta_j$: $Q(2) = 1.86$, $p = 0.39$

Test of $\theta = 0$: $z = -3.00$, $p = 0.00$

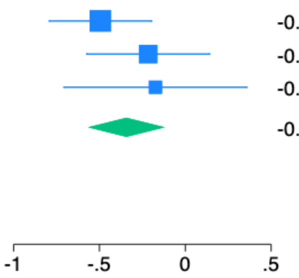

Weight

Baseline

Study	AKI N	AKI Mean	AKI SD	No AKI N	No AKI Mean	No AKI SD	Hedges's g with 95% CI	Weight (%)
Zappitelli	73	-0.32	1.13	86	-0.18	2.13	-0.08 [-0.39, 0.23]	43.37
Zappitelli	69	-0.49	2.15	207	-0.16	4.63	-0.08 [-0.35, 0.19]	56.63
**Overall**							-0.08 [-0.28, 0.13]	

Heterogeneity: $\tau^2 = 0.00$, $I^2 = 0.00\%$, $H^2 = 1.00$

Test of $\theta_i = \theta_j$: $Q(1) = 0.00$, $p = 0.99$

Test of $\theta = 0$: $z = -0.76$, $p = 0.45$

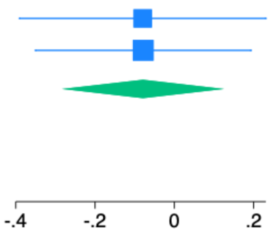

3 Months – <3 Years

Study	AKI N	AKI Mean	AKI SD	No AKI N	No AKI Mean	No AKI SD	Hedges's g with 95% CI	Weight (%)
Zappitelli (3m)	71	-0.48	1.06	85	-0.12	2.09	-0.21 [-0.53, 0.10]	37.49
Zappitelli (12m)	65	-0.26	1.10	84	-0.05	2.11	-0.12 [-0.44, 0.20]	35.66
Zappitelli (3y)	50	-0.35	1.18	62	0.07	1.90	-0.26 [-0.63, 0.12]	26.86
**Overall**							-0.19 [-0.38, 0.00]	

Heterogeneity: $\tau^2 = 0.00$, $I^2 = 0.00\%$, $H^2 = 1.00$

Test of $\theta_i = \theta_j$: $Q(2) = 0.32$, $p = 0.85$

Test of $\theta = 0$: $z = -1.94$, $p = 0.05$

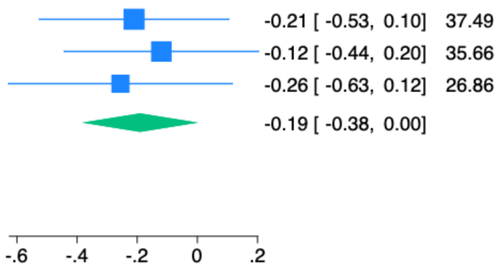

>3 Years

Study	AKI N	AKI Mean	AKI SD	No AKI N	No AKI Mean	No AKI SD	Hedges's g with 95% CI	Weight (%)
Zappitelli (6y)	69	0.22	1.55	207	0.01	1.39	0.15 [-0.13, 0.42]	51.24
Zappitelli (7.5y)	46	0.23	1.54	121	0.26	1.38	-0.02 [-0.36, 0.32]	33.18
Zappitelli (11y)	21	0.26	1.77	59	0.31	1.87	-0.03 [-0.52, 0.47]	15.58
**Overall**							0.06 [-0.13, 0.26]	

Heterogeneity: $\tau^2 = 0.00$, $I^2 = 0.00\%$, $H^2 = 1.00$

Test of $\theta_i = \theta_j$: $Q(2) = 0.72$, $p = 0.70$

Test of $\theta = 0$: $z = 0.64$, $p = 0.52$

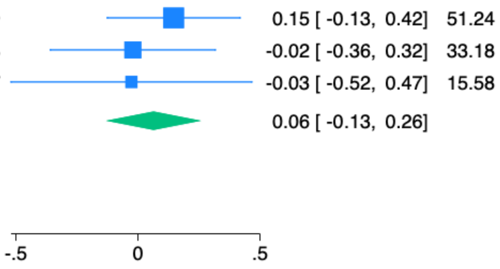

Supplement: Supplementary file 2 — Supplementary file1 (PDF 1.49 MB) [file 467_2025_6801_MOESM2_ESM.pdf]
